# The Potential of Ancient Sicilian Tetraploid Wheat in High-Quality Pasta Production: Rheological, Technological, Biochemical, and Sensory Insights

**DOI:** 10.3390/foods14122050

**Published:** 2025-06-11

**Authors:** Rosalia Sanfilippo, Nicolina Timpanaro, Michele Canale, Salvatore Moscaritolo, Margherita Amenta, Maria Allegra, Martina Papa, Alfio Spina

**Affiliations:** 1 Council for Agricultural Research and Economics (CREA), Research Centre for Cereal and Industrial Crops, Corso Savoia, 190, 95024 Acireale, Italy; michele.canale@crea.gov.it (M.C.); alfio.spina@crea.gov.it (A.S.); 2 Council for Agricultural Research and Economics (CREA), Research Centre for Olive, Fruit and Citrus Crops, Corso Savoia, 190, 95024 Acireale, Italy; margherita.amenta@crea.gov.it (M.A.); maria.allegra@crea.gov.it (M.A.); martina.papa@crea.gov.it (M.P.); 3 Council for Agricultural Research and Economics (CREA), Research Centre for Cereal and Industrial Crops, S.S. 673 km 25.200, 71122 Foggia, Italy; salvatore.moscaritolo@crea.gov.it

**Keywords:** tetraploid wheats old landraces, organic semolina, technological performance, antioxidant activity, phenolic compounds, high-quality pasta, cooking quality, sensory analysis

## Abstract

This study evaluated the potential of three ancient Sicilian tetraploid wheat genotypes—‘Margherito’, ‘Perciasacchi’, and ‘Russello’—for organic pasta production, compared to the national variety ‘Cappelli’. Significant variations in particle size distribution were found, with ‘Russello’ exhibiting the highest proportion of fine particles and the greatest protein content (14.30% d.m.). ‘Perciasacchi’ displayed the highest gluten index (81.26%). ‘Margherito’ and ‘Cappelli’ had the highest antioxidant activity, with ‘Margherito’ showing elevated levels of lutein and total carotenoids. Rheological analysis revealed differences in dough properties. ‘Perciasacchi’ exhibited the highest dough stability and P/L ratio (6.57), whereas ‘Russello’ showed the lowest values for both. Additionally, ‘Russello’ had lower consistency (12 B.U.), reduced gel stability, and limited water retention in the visco-amylographic analysis. Pasta quality was evaluated based on cooking time, water absorption, and texture. Cooking time ranged from 10 to 12 min, with ‘Russello’ and ‘Margherito’ showing lower water absorption. Texture analysis indicated that ‘Margherito’ pasta was the least firm, while ‘Russello’ showed the greatest loss of consistency when overcooked. From a sensory perspective, ‘Russello’ had lower firmness, but a stronger semolina flavor and surface roughness. ‘Cappelli’ had the most intense cooked pasta odor, while ‘Perciasacchi’ was the hardest and least sticky, though less flavorful. The results support the use of ancient tetraploid wheat genotypes as valuable resources for sustainable, high-quality pasta production.

## 1. Introduction

Wheat (*Triticum* spp.) cultivation has a millennia-long history, characterized by extensive genetic and agronomic diversity. Over time, selective breeding and adaptation to different climatic conditions have led to the diversification of wheat varieties. Today, *Triticum aestivum* subsp. *aestivum* (bread wheat) accounts for the majority of the world’s production due to its high yield potential and technological versatility, making it the primary choice for bread and baked goods. In contrast, *Triticum turgidum* subsp. *durum* and subsp. *turanicum* (tetraploid wheat) represent a smaller share of global wheat production and thrive in Mediterranean-like environments due to their tolerance to heat and drought stress.

Despite its limited production, durum wheat has a strategic relevance in the production of high-quality dry pasta due to its unique physico-chemical properties [1]. Durum wheat semolina is appreciated for its coarse grain size, high protein content, gluten strength and intense yellow color, mainly due to carotenoids, especially lutein, which contributes significantly to the visual appeal of dry pasta.

Italy holds a leading position in both pasta production and per capita consumption, supported by its deep-rooted culinary tradition and an advanced durum wheat processing industry [2]. Approximately 70% of Italian durum wheat cultivation is concentrated in the southern regions, such as Sicily, where suitable soil and climate conditions support optimal growth [3]. These regions are also repositories of rich agro-biodiversity, preserving both modern varieties and ancient landraces characterized by distinctive bio-morphological traits and specific quality attributes [4].

The cultivation of ancient wheat varieties has experienced a period of decline, mainly due to their generally lower yield potential compared to modern varieties, late maturity, and increased susceptibility to lodging [5,6]. In addition, agricultural policies throughout the 20th century have prioritized high-input systems.

However, recent shifts in consumer awareness towards health, sustainability, and product traceability have led to renewed interest in these ancient wheat varieties. These genotypes exhibit distinctive traits [7], including high adaptability to organic and low-input farming systems, increased resilience to both biotic and abiotic stresses, and unique compositional profiles—often rich in dietary fiber, phenolics, and micronutrients. These attributes make them particularly attractive for the development of more nutritious and sustainable food products [8,9].

The renewed interest in these ancient varieties is driven not only by an increased interest in preserving agricultural biodiversity, but also by their revaluation in modern food production, especially in the production of high-quality pasta.

In this context, Cappelli, developed by the Italian geneticist Nazareno Strampelli, is an ancient durum wheat variety with a long history and a significant contribution to the genetic background of many modern varieties [10]. Locally, Sicily has a rich heritage of durum wheat landraces such as Russello, Perciasacchi, and Margherito, cultivated by custodian farmers within traditional agro-ecosystems. These genotypes are gaining ground due to their adaptability to organic practices, their distinctive sensory profiles, and their promising suitability for the production of high-quality pasta, in line with current consumer preferences [11,12].

Their use could support the dual objectives of preserving agro-biodiversity and the environment, thanks to the lower use of chemical inputs and meeting modern consumer expectations for clean-label, authentic, nutritious, and environmentally sustainable foods [13,14].

From a nutritional perspective, these genotypes generally have more favorable profiles than modern cultivars, with higher levels of phenolic compounds, vitamins, minerals, and other bioactive compounds, which can improve the functional quality of dry pasta and contribute to its health-promoting properties [15,16].

Despite growing interest in sustainable food systems, scientific research on pasta production using ancient durum wheat varieties remains limited. Current studies mostly focus on the ‘Cappelli’ variety, and information on other traditional landraces, particularly those from specific regions such as Sicily, is limited.

Additionally, previous research has predominantly focused on the morphological, physiological, and genetic traits of these genotypes [7,11,17,18], but their technological potential, especially regarding semolina quality and processing behavior, has largely been overlooked. Most existing data are limited to bread-making applications, leaving a significant knowledge gap regarding their suitability for dry pasta production [19,20,21].

The present study breaks new ground as the first to investigate the pasta-making performance of ancient Sicilian tetraploid wheat genotypes. By focusing on three genotypes—‘Margherito’ (syn. Bidì), ‘Perciasacchi’ (syn. Farro Lungo), and ‘Russello’ (syn. Preziosa)—and comparing them with the well-studied Cappelli variety, it addresses a significant knowledge gap and contributes to a deeper understanding of their technological value. All genotypes were cultivated under organic conditions in Sicily. The analysis covers physico-chemical and biochemical properties, rheological behavior, and technological performance. Additionally, the quality of pasta produced is evaluated through key indicators, including cooking performance, and sensory attributes.

The ultimate goal is to explore the potential of these ancient Sicilian durum wheat varieties for producing high-quality pasta, reinforcing their role in preserving the cultural and genetic heritage of Sicilian cereal crops within sustainable agri-food systems.

## 2. Materials and Methods

### 2.1. Plant Materials and Environmental Conditions

Among the twenty-four Sicilian local varieties of tetraploid wheat registered in the National Register of Conservation Varieties, two durum wheat (*Triticum turgidum* subsp. *durum*) genotypes (Margherito and Russello) and one *T. turgidum* subsp. *turanicum* (Perciasacchi) were identified as particularly suitable for pasta-making and were compared with Cappelli, the ancient national reference variety.

The field trial was carried out during the 2022/23 growing season on ‘Lorìa 75’ farm, located in ‘Tumarrano’ (Lat. 37.625898–Lon. 13.727°-336 m a.s.l.) (Cammarata, Agrigento, Italy), on a clay soil, to evaluate the main quality of the grain. The trial environment is typical of inland hilly Sicily (meso-thermo-Mediterranean climate), with long, dry summers and cool winters, with very limited snowfall and irregular rainfall. Genotypes were laid out in the field in large 1500 m^2^ plots, according to a randomized-block experimental design with three replicates, following organic wheat agronomic management.

Grain harvesting took place in mid-June 2023, when the grain moisture fell below 14%, in order to prevent the development of mold, fungal growth, and other phytosanitary issues, such as mycotoxin production.

### 2.2. Determination of Grain Characteristics

The grain from each genotype, originating from the three field replicates, was bulked, and the main commercial quality parameters were determined: moisture content, thousand kernel weight (TKW), test weight (TW), and grain defects, including starchy and shrunken kernels.

TKW was calculated by weighing 8 sub-samples of 100 kernels, and the average weight was related to 1000 kernels. Moisture and TW were determined with an Infratec 1241 Grain Analyser (Foss Tecator, Hillerød, Denmark); in particular, TW was detected by the Test Weight Module (TWM) installed under the device. The percentages of starchy and shrunken kernels were visually estimated from representative 30 g sub-samples. Kernels were visually classified into fully vitreous and non-vitreous (i.e., containing at least two starchy spots), and the proportion of non-vitreous kernels was expressed as a percentage of the total kernels.

### 2.3. Milling Process

Grains of Margherito, Russello, Cappelli (*Triticum turgidum* subsp. *durum*), and Perciasacchi (*Triticum turgidum* subsp. *turanicum*) were milled using an assembled cylinder mill with a processing capacity of 2.5 t/h, incorporating components from various manufacturers (Bühler, Uzwil, Switzerland; Golfetto, Padua; Negretti, Brescia, Italy). The milled product was fractionated with a plansichter (Sicom, Naples, Italy) and further refined with a triple semolina purifier equipped with six sieves (Sicom, Naples, Italy).

Granulometric analysis of the semolina was conducted by dry sieving with a Retsch AS 200 granulometric tower (Haan, Germany), equipped with the following superimposed sieves: 500, 350, 250, 160 µm, and <160 µm, arranged in decreasing aperture size. The particle size fraction retained on each sieve after 10 min of sieving, based on 50 g of sample, was expressed as a percentage.

### 2.4. Physico-Chemical Features of Semolina and Pasta

The moisture content of semolina and pasta was analyzed by oven-drying (Memmert, Milan, Italy) at 103 °C to a constant weight, following the AOAC method 935.25 [22]. The results for semolina were expressed as percentage of dry matter (% d.m.).

The quality of the semolina was evaluated by means of main chemical and technological analyses. Ash content was obtained following the ISO 2171:2007 method [23]. Protein content was determined using ICC Standard No. 167 [24], with a nitrogen-to-protein conversion factor of 5.7 applied for durum wheat. Gluten analysis was performed using a Glutomatic apparatus, a centrifuge 2015, and a Glutork 2020 (Perten Instruments AB, Huddinge, Sweden) to determine wet and dry gluten and to calculate the gluten index, according to ICC Standard No. 158 [25] and the AACC 38-12.02 method [26], respectively. Wet gluten was separated by centrifugation under standardized conditions using a specially designed sieve. The ‘B-fraction’ refers to the portion of wet gluten passing through the sieve. A higher B-fraction indicates lower technological quality of the gluten [27].

The color of the semolina, raw pasta, and cooked pasta was evaluated using a colorimeter (CR 200, Minolta, Osaka, Japan) in triplicate for each sample, both before and after cooking. The CIELab model with illuminant D_65_ was applied to measure the following parameters: L*, lightness, ranging from 0 (black) to 100 (white); a*, red index (from green to red); and b*, yellow index (from blue to yellow) [21].

### 2.5. Contents of Carotenoids, Total Polyphenols, and Antioxidant Activity (ORAC Assay)

The carotenoids were extracted using a modified literature procedure, first detailed by Vignolini et al. [28]. In particular, 10 g of semolina was extracted with 100 mL acetone and cold-sonicated for 30 min. The sample was centrifuged for 5 min at 5000 rpm, the supernatant evaporated to dryness with a Rotavapor, and the residue was dissolved in 5 mL of acetone. The extracts were used for HPLC-DAD analysis.

Carotenoid content was determined using a Waters Alliance liquid chromatography system equipped with a quaternary pump, a photodiode array detector, an autosampler, and Empower Manager software (v. 3.8.1). The carotenoid separation was carried out using a C30 carotenoid column (250 × 4.6 mm i.d., 5 μm) (YMC Inc., Wilmington, NC, USA). The mobile phases were mixtures of water and acetonitrile (70:30 *v*/*v*; channel A) and methanol–methyl tert-butyl ether (50:50 *v*/*v*; channel B). The eluent composition changed from 20% to 47% of channel B in 10 min, from 47% to 57% of channel B over the next 10 min, and from 57% to 100% of channel B over 20 min, followed by an isocratic hold at 100% channel B for 13 min. The total run time was 53 min. After each analysis, the column was re-equilibrated to the starting conditions. The running flow rate was 0.4 mL/min, the quantity of injected sample was 20 µL, and all carotenoids were monitored at 450 nm.

Total polyphenol content was determined by the Folin–Ciocalteu spectrophotometric method [29], using a UV-Vis spectrophotometer (Model Cary 100 Scan, Varian, Palo Alto, CA, USA). An aliquot of 10 g of ground semolina was extracted in 50 mL of a 70:30 ethanol/water solution (EtOH/H_2_O) adjusted to pH 3.2 with formic acid; it was extracted for 24 h in the dark on a rocking shaker. The ethanolic solution was centrifuged at 1400 rpm for 5 min, filtered, and then removed. A suitably diluted aqueous aliquot of the solution (1 mL) was mixed with 5 mL of commercial Folin–Ciocalteu reagent (previously diluted 1:10 *v*/*v* with water) and 4 mL of 7.5% sodium carbonate solution. The mixture was incubated in the dark at room temperature for 2 h, then read spectrophotometrically at 740 nm, and the concentration of total phenolics was expressed as mg/100 g gallic acid.

Antioxidant activity was determined using the ORAC assay, as described by Cao, Alessio, and Cutler (1993) [30] and improved by Ou, Hampsch-Woodill, and Prior (2001) [31], with some modifications. Briefly, measurements were performed on a Wallac 1420 Victor III 96-well plate reader (EG & Wallac, Turku, Finland) equipped with fluorescence filters (excitation 485 nm, emission 535 nm). Fluorescein (116 nM) was the target molecule for free radical attack by AAPH (153 mM) as a peroxyl radical scavenger. The reaction was performed at 37 °C pH 7.4, with Trolox (1 μM) as the control standard and 75 mM phosphate buffer (pH 7.4) as the blank. All solutions were freshly prepared before analysis.

An aliquot of 10 g of ground semolina was extracted in 50 mL of a 70:30 solution (EtOH/H_2_O), corrected to pH 3.2 with formic acid, for 24 h in the dark on a rocking shaker. The ethanolic solution was centrifuged at 1400 rpm for 5 min, filtered, and then removed. The samples were subsequently diluted with phosphate buffer (1:100, *v*/*v*).

### 2.6. Rheological and Technological Tests

From a rheological and technological point of view, several methods were used for dough analysis. Dough samples were tested with a Brabender Farinograph (Duisburg, Germany) to assess the water absorption of semolina, dough development time, and dough stability, following the AACC 54-21 method [26].

An Alveograph NG instrument, equipped with the Alveolink NG V1.04/99 software (Tripette et Renaud, Chopin Technologies, Villeneuve la-Garenne, France), was used to measure the deformation energy (W) and the tenacity/extensibility ratio (P/L) of the samples, according to the UNI 10453 method [32].

Mixograph analysis was performed to assess the mixing time and peak dough height. This analysis employed a National MFG Mixograph Co. instrument (Lincoln, NE, USA), generating a mixograph curve in accordance with the AACC method 54-40.02 [26].

The amylase activity of the semolina samples was determined using a Falling Number 1500 apparatus (Perten Instruments AB, Huddinge, Sweden), as specified in ISO 3093 [33].

The amylographic analysis recorded starch granule behavior during heating and cooling in water. It was performed using a Micro-Visco Amylograph (Brabender, Duisburg, Germany), suitable for analyzing small quantities of samples subjected to heating and subsequent cooling. This analysis is performed on 15 g of semolina (14% moisture) suspended in 100 mL of distilled water, which can be subjected to different thermal profiles [34]. The AACC 22-12 method [26] is generally used for the analysis of flour intended for bread, while the AACC 54-60.01 protocol [26], adopted by the Mixolab Chopin+, is used for the analysis of semolina intended for pasta, as it is more suited to the cooking times of pasta. The applied thermal profile is shown in Table S1. Specifically, curves like those shown in Figure S1 are obtained, with time (in minutes) plotted on the x-axis and both viscosity (in Brabender units, B.U.) and temperature (in °C) on the y-axis.

First, the suspension was kept at 30 °C under stirring at 250 rpm. It was then heated to 90 °C at a rate of 4 °C/min for 15 min (phase 1), held at 90 °C for 7 min (phase 2), cooled to 50 °C at a rate of −4 °C/min for 10 min (phase 3), and finally kept at this temperature for 5 min (phase 4) [35].

For each point on the curve, the time t (min:ss), the temperature T (°C), and the viscosity (B.U.) were recorded. Details of each point are provided in Table S2.

### 2.7. Pasta-Making Process

The four pasta samples were produced in an artisanal pasta production plant (Dominioni, Food Machinery Division EL.MA, Alseno, Piacenza, Italy) with an hourly production capacity of between 600 and 850 kg per day. After thorough mixing of the ingredients, the pasta was extruded through bral dies (bronze and aluminium) (Turconi, Colverde, Como, Italy). These dies were chosen instead of Teflon dies because they give the pasta a much rougher and more porous surface. This characteristic is crucial in improving the pasta’s ability to enhance sauces and for condiment retention [36]. Next, the pasta was subjected to a drying process using a static dryer model E25 (Dominioni, Food Machinery Division EL.MA, Alseno, Piacenza, Italy) at a controlled, relatively low temperature between 37 and 41 °C. The drying process lasted between 60 and 80 h, allowing a gradual and homogeneous reduction in the moisture content to ensure a constant and optimal quality of the final product.

### 2.8. Pasta Cooking Quality and Sensory Analysis

The cooking quality of the pasta was assessed following the ISO 7304-1 standard [37]. The optimum cooking time (OCT) is defined as the time required for the disappearance of the continuous white line visible in the center of a strand of pasta. Conventionally, the white line is considered to have disappeared when it is only visible as a series of dots. This parameter was determined by placing a 100 g sample of each type of pasta in 1500 mL of distilled water. During cooking, the spaghetti was stirred three times for 10 s with a fork. Individual strands of spaghetti were removed every 30 s and pressed between two Plexiglas crushing plates until the central white core was no longer visible.

The water absorption index (WAI) measures the ability of pasta to retain water during cooking, a factor that significantly affects texture and mouthfeel. It was analyzed by comparing the weight of the pasta before and after cooking, as reported by Bokić et al. (2022) [38]. The following formula was applied to calculate WAI:

$$WAI(\%)=\frac{Weight\;of\;cooked\;pasta-Weight\;of\;raw\;pasta}{Weight\;of\;raw\;pasta}*100$$

A caper was used to analyze the thickness of the spaghetti. This measurement on raw pasta, particularly in long formats such as spaghetti, was essential to evaluate its stability during the drying process, while measurements on cooked pasta assessed swelling and therefore the degree of consistency.

The spaghetti was also cooked for 15 min, beyond the OCT, to assess its overcooking performance/tolerance.

The sensory analyses were carried out at the sensory analysis laboratory of CREA (Acireale, Italy), set up in accordance with the ISO 8589:2007/Amd 1:2014 standard [39]. The laboratory was equipped with specific software for the acquisition of sensory data (Smart Sensory box, Smart Sensory Solutions S.r.l., Sassari, Italy).

All assessors were selected and trained in accordance with ISO 8586:2023 [40]. Sensory descriptive analysis was carried out both on dried and cooked pasta samples by a panel of 10 trained sensory assessors (6 females and 4 males aged 25–55 years) in the sensory laboratory. Each judge received 15 g of cooked pasta in a covered dish. The order of sample presentation was balanced and randomized between the judges. Each sample was presented with a 3-digit code automatically generated by Smart Sensory box software. A 9-point scale was employed, ranging from 1 (the absence of sensation) to 9 (the highest intensity) for each attribute. Water at room temperature was used for palate cleansing. In this study, commercial pasta products on the market were used as reference standards during the training of the judges.

For the sensory analysis of pasta, the ISO 7304-1:2016 and ISO 7304-2:2008 standards were used [37,41]. These standards establish a method for the sensory assessment of cooking quality, as well as the evaluation of firmness, liveliness, and starch release through the manual manipulation of pasta shaped as long spaghetti, as in this study. These latter parameters were determined on cooked pasta by assigning scores ranging from 10 to 100 (Table 1). Firmness refers to the resistance felt when cutting the pasta strands between the teeth; liveliness is the ability of one strand of spaghetti to slide smoothly over another and is closely related to its degree of stickiness; starch release denotes the amount of surface-level starch released during cooking, detectable through tactile evaluation.

Quantitative Descriptive Analysis (QDA) was applied to obtain the sensory profile of the developed pasta. In addition, ANOVA was used to differentiate the samples, while a Spider plot was developed to analyze the sensory profile of the samples, representing the mean scores of the sensory analysis.

Ten sensory descriptors were evaluated (Table S3) to create the sensory profile of the pasta [42].

### 2.9. Texture Analysis

The evaluation of the texture of cooked pasta was carried out according to the specifications of the AACC method 66-50 (2000) [26], using a TA.XT plus texture analyzer equipped with a 25 kg load cell (Stable Micro System, Godalming, UK) and fitted with a standard knife probe (TA47). The setting parameters used for the analyses were as follows: test mode = compression; pre-test speed = 1.0 mm/s; test speed = 2.00 mm/s; post-test speed = 10.0 mm/s; target mode = distance; force = 100.0 g; cutting blade setting distance = 4.90 mm (0.10 mm from base); % cut = 100.0%. Specifically, the analyzer generates a curve (Figure S2) with the following parameters: the ‘work’ (total area—Kg.s), ‘force’ (N), and ‘time’ (s) required to cut 5 strands of cooked spaghetti placed parallel to each other at a distance of 1 cm were measured.

### 2.10. Statistical Analysis

All data (mean ± standard deviation) were subjected to one-way analysis of variance (ANOVA) using Statgraphics^®^ Centurion XVI (Statpoint Technologies, The Plains, VA, USA) software.

A principal component analysis (PCA) was performed on the entire dataset, encompassing the physical and chemical characteristics of semolina, dough, and both raw and cooked pasta from four traditional varieties of durum wheat. PCA was performed using PAST, PAleontological STatistics software package, 2011 [43].

All analyses were performed in triplicate.

## 3. Results and Discussion

### 3.1. Grain Characteristics and Relative Defects

The grain characteristics and relative defects of the evaluated genotypes are reported in Table 2. Test weight and thousand kernel weight were significantly higher in the *Triticum turgidum* subsp. *turanicum* Perciasacchi (83.6 kg/hL and 62.0 g, respectively). As expected, turanicum wheat had larger kernels compared to durum wheat. The historical variety Cappelli also showed elevated values for both test weight and thousand-kernel weight (82.6 kg/hL and 50.1 g, respectively), while Margherito displayed lower values for both parameters, with test weight below 80 kg/hL and thousand-kernel weight below 50 g. These results agree with those reported by other authors [19]. A high degree of variability was observed in the incidence of starchy kernel defects, which is characterized by an opaque, floury endosperm resulting from impaired starch accumulation [44]. For the Russello genotype, nearly one-quarter of the kernels were affected by the starchy kernel defect, in contrast to an average incidence of only 3.6% in the other genotypes. Notably, this low frequency contrasted with the findings reported by Fiore et al. [19], who observed higher levels of starchy kernel expression in the Cappelli and Margherito genotypes under different environmental conditions. These results confirmed the high variability of the trait and suggested that the expression of this defect is strongly influenced by genotype–environment interactions, particularly heat stress and water deficit during the grain filling stage, which disrupt normal starch biosynthesis and deposition.

Regarding shrunken kernels, the examined genotypes showed a low percentage of this defect. Margherito had the highest statistically significant percentage (almost 5%), while Cappelli and Russello showed lower incidences (3.31% and 1.80%, respectively). Notably, Perciasacchi was virtually free from this kernel defect. The black pointed kernel defect was not found.

### 3.2. Physico-Chemical Characterization of Semolina

Studies conducted using the particle size tower showed statistically significant differences between the samples (Table 3). The distribution of coarse particles (500 µm fraction) was quite low in all samples, with significant statistical differences. Specifically, Margherito and Cappelli showed significantly lower values (0.67% and 0.72%, respectively), suggesting a finer grain size than Russello and Perciasacchi (1.02% and 0.94%, respectively). Perciasacchi showed a broader distribution, having the highest percentage of particles in the 350 µm size range (32.63%), while Russello had the lowest (9.28%). Margherito and Cappelli exhibited intermediate values.

The 250 µm fraction was significantly higher in Margherito (22.68%) compared to the other samples, while Perciasacchi and Cappelli exhibited similar distributions, according to the statistical analysis.

Particles of 160 µm size were more abundant in Margherito and Cappelli (29.12% and 29.05%, respectively) than in Russello and Perciasacchi.

Finally, very fine particles (<160 µm) were significantly observed in Russello (53.84%), which could indicate a finer texture than the other samples, which showed statistically lower values. A possible explanation may lie in grain defects. Russello exhibited a high incidence of starchy kernels, affecting about one-quarter of the grains. This condition increases grain friability, leading to the production of a finer, almost powdery flour.

The degree of grinding, which defines particle size, has a strong influence on technological and quality parameters. Smaller particles have a larger contact surface area and lack bran layers, positively affecting parameters such as water absorption, dough extensibility, and the texture of finished products [45].

The dry matter content fluctuated around values between 87.33% and 88.18%, showing significant statistical differences between the samples. For ash, there were slight but statistically significant differences between the samples, particularly between Margherito (1.28%) and Russello (1.19%).

The ash content was slightly higher than the limit set by the Italian law for semolina (maximum 0.90%). This was due to a shrunken kernel defect, which required a reduction in the distance between the ‘undressing’ cylinders after kernel breaking. Consequently, less bran was removed, leaving a finely milled fraction in the semolina. Furthermore, these genotypes tend to have higher mineral content, which contributes to the increased ash levels in the milled product.

The protein content, expressed in dry matter, ranged from 13.54% (Perciasacchi) to 14.30% (Russello), both above the minimum limit of 10.50% set by Italian legislation [46]. The results agree with those of other authors’ studies conducted on Perciasacchi, Russello, and Cappelli [20].

The dry gluten percentage was highest in Russello (9.85%) and Margherito (10.02%), while Perciasacchi and Cappelli showed lower values in line with their protein content. The dry gluten/protein ratio indicated that the optimal value was recorded in Margherito (70.98% d.m.).

In terms of gluten index, Perciasacchi showed the highest value (81.26), suggesting a higher gluten quality than the other samples. This was particularly notable given its lower protein content, which may indicate greater gluten functionality than protein concentration. On the contrary, the lowest gluten index was found in Russello (50.95).

As indicated by other authors [20,47], the quality of wheat is not only determined by the quantity of protein, but also by its quality. A high gluten quality (gluten index) above 80 reflects an excellent protein quality of the semolina, and is a critical factor in the selection of raw materials for both bread and pasta production [48].

Color is a key factor in consumer preference and purchase decisions. In particular, the amber-yellow color is a distinctive feature of pasta and bread, identifying them and enhancing their attractiveness. The analysis of the colorimetric parameters of semolina (Table 3) highlighted a certain variability among the different genotypes. Russello had the highest lightness index (88.55 L*), significantly higher than the other samples, which did not differ significantly among themselves. All values are in line with the results in the literature [19].

The Russello sample showed lower redness than in compared to the other samples, Perciasacchi had the highest value, indicating a less pronounced green tone. Margherito and Cappelli fell in the middle, with no significant differences between them or with Perciasacchi.

The yellow index, linked to the carotenoid content in raw materials, enhances the appeal of pasta and bakery products, influencing their commercial value. The yellow index results revealed statistically significant differences. Perciasacchi had the highest value (17.30 b*), reflecting the most intense yellow color. Margherito and Cappelli had slightly lower but comparable values, while Russello, with the lowest value (11.54 b*), was significantly less yellow. The presence of high levels of starchy kernel defects in Russello grain could explain the reduced yellow index of its semolina, since these areas generally have lower pigment concentrations. This value of Russello was also lower than that reported by Fiore et al. [19], while other samples aligned.

These results match Ruisi et al. [20], with Perciasacchi semolina being more yellow than Russello and Cappelli, despite a generally lower yellow index in this study.

### 3.3. Rheological and Technological Features of Semolina

From a rheological and technological perspective, farinograph analysis revealed substantial variability in dough development time and stability across the genotypes studied (Table 4). In particular, Russello exhibited the shortest development time (2.50 min), in stark contrast to Cappelli, which required the longest (4.85 min). Dough stability, as measured by the farinograph, also demonstrated a marked statistical variability. For example, Russello and Margherito had relatively low stability values of 2.75 and 5.65 min, respectively. In contrast, Perciasacchi and Cappelli stood out for their significantly higher stability, maintaining optimal dough characteristics for 16.95 and 13.40 min, respectively.

Water absorption showed no statistically significant differences between the genotypes, indicating that this characteristic was not influenced by genetic variation. However, the alveographic indices displayed considerable variability, shedding light on the mechanical properties of the dough. Regarding the W parameter, which reflects the dough strength, Perciasacchi and Cappelli exhibited the highest values, 166.33 (10^−4^ × J) and 162.33 (10^−4^ × J), respectively, highlighting their strong dough-making properties and potential for pasta-making. In contrast, Margherito and Russello yielded weaker doughs with lower W values.

The P/L ratio, a critical measure of the balance between dough elasticity and extensibility, further highlighted the differences between the varieties. The Cappelli genotype recorded the highest P/L ratio (7.23), closely followed by Perciasacchi (6.57), indicating a dough profile predominantly characterized by tenacity. On the other hand, Margherito (3.43) and Russello (1.24) exhibited significantly lower P/L ratios. The predominance of fine particles (Table 3), especially for Russello (53.84%), could explain this. Fine particles tend to overhydrate quickly, softening the dough and increasing extensibility, which would lower the P/L ratio.

The high P/L values recorded for Perciasacchi (6.57) and Cappelli (7.23) reflected doughs with high tenacity (P) relative to extensibility (L).

In the semolina samples, amylase activity was rather low, as shown by the high values of the falling number (FN), which is inversely proportional to amylase activity. The highest values of FN were recorded for Perciasacchi (524.50 s) and Cappelli (539.50 s), which showed a reduced amylase activity.

The mixograph analysis, carried out to evaluate the behavior of the dough during mixing, gave results that appear to agree with the farinograph data. Specifically, the mixograph curve showed a longer mixing time for the Cappelli sample (5.14 min) than for the others, with no statistically significant differences in the peak dough height.

Based on the results presented so far, a correlation can be hypothesized between particle size distribution (Table 3) and the overall quality of the semolina, particularly in relation to its technological properties [49]. Although there were no statistically significant differences in water absorption on the farinograph, it could be hypothesized that the slight variations in water uptake and alveographic results could be caused by differences in particle size distribution between the samples. It appeared fairly uniform in the Perciasacchi, Cappelli, and Margherito samples. Only Russello showed a deviation, with almost 54% of fine particles being <160 µm. As can be seen from Table 4, the technological data showed some variability. In particular, the farinographic results for Russello show a rapid dough development (2.50 min) compared to the other samples. This could be due to the greater proportion of fine particles (<160 µm), which tend to absorb water more quickly due to a larger surface area, leading to a faster hydration process. This would lead to the development of a weak, soft, and more extensible dough (higher L-value), thus reducing the P/L ratio.

At the same time, Perciasacchi has a longer development time and higher W and P/L, as well as lower water absorption. This outcome may once again be attributed to the greater proportion of 350 µm particles, which likely reduce water uptake.

### 3.4. Contents of Carotenoids, Total Polyphenols, and Antioxidant Activity

The semolina was also analyzed for its total polyphenol content and antioxidant activity using the ORAC assay (Table 5). Total carotenoid content was also considered. As reported in previous studies [28,50], lutein was the main carotenoid identified in *Triticum* spp.

In the present study, the lutein content showed a significant difference (*p* ≤ 0.001) between Margherito and the other samples, which was also confirmed by the results for total carotenoids. The Margherito landrace featured a major content of lutein (10.73 mg/Kg) and also of total carotenoids (11.24 mg/kg). In contrast, Russello had the lowest level of total carotenoids. This could explain the lower yellow index recorded for Russello compared to the other samples, since these pigments are key contributors to food color.

Still regarding the polyphenolic content, the Cappelli cultivar was richest in polyphenols (15.84 mg GAE/100 g), although no statistically relevant differences were observed between the samples. The antioxidant nature of these compounds also explained the highest antioxidant activity recorded by this sample (39.33 µmol TE/g) compared to the other genotypes (Russello and Perciasacchi).

The values agree with the findings by other authors [28] regarding polyphenol content, antioxidant activity, and carotenoids in semolina obtained from stone-ground wheat.

### 3.5. Micro Visco-Amylograph Parameters

**Figure 1:**
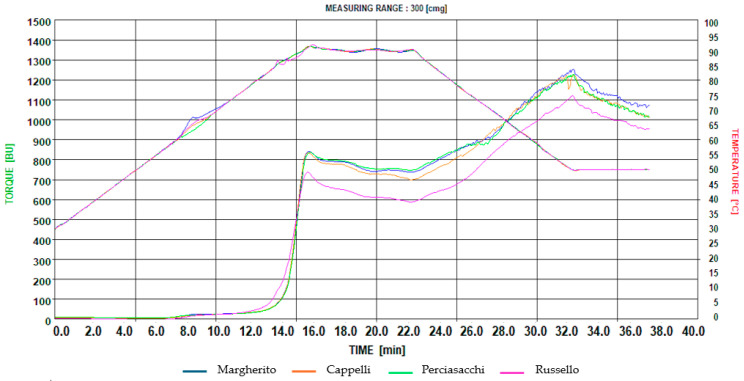
Pasting characteristics of the four spaghetti samples.

The visco-amylographic profiles of the four semolina samples are shown in Figure 1. The results of this analysis (Table S4) showed that the starting point of gelatinization of the most accessible starch granules (point A) occurred at 63 °C (±1.02) for all cultivars at 08:03 min (±00:07), with slightly different consistency values ranging from Russello (12 B.U.) to Perciasacchi (17 B.U.). On the other hand, the values related to the rapid increase in the gelatinization of all starch granules (point C) differed, with the Russello cultivar first showing maximum starch granule permeability at 12:00 min at 78 °C, compared to the other cultivars, which showed changes at higher temperatures (82.4 ± 1.0) at 13:07 min (±00:18).

The phase of complete gelatinization of the starch granules (point B) and the phase of stability of the hot gel (point D) showed no significant differences between the genotypes in terms of time or gelatinization temperatures, which occurred at 91.3 °C (±0.2) at 15 min, 40 s’ (±11 s), and at 90.0 °C at 22:00 min, respectively. Russello differed, showing lower values of 741 B.U. at point B and 591 B.U. at point D, compared to average values of approximately 841 B.U. (±11) and 730 B.U. (±10) at both points for the other cultivars.

The consistency of this variety was lower, showing a more fluid gel that binds a small amount of water. Consequently, the gel stability value in the hot phase for this variety, calculated as the difference between the two points (breakdown viscosity), showed lower gel stability in the hot phase, with a weakening of the gel to 151 B.U. compared to the other varieties, which recorded an average value of 112 B.U. (±17).

After cooling the gel from 90 to 50 °C, all the genotypes showed an increase in their consistency due to the reorganization of the starch structure, which becomes more compact and therefore less digestible. At point E (50 °C), Russello reached a value of 1114 B.U., lower than the average of the other cultivars, which showed similar values, averaging 1240 B.U. (±20). Also, in the subsequent phase related to gel stability at 50 °C (point F), Russello showed the lowest value (958 B.U.) compared to the average values of the other three cultivars of 1042 B.U. (±23), confirming a lower water retention capacity for this cultivar.

### 3.6. Cooking Quality and Pasta Characterization

The evaluation of the cooking quality of pasta, as shown in Table 6, highlighted significant differences between the four pasta samples.

The optimum cooking time (OCT) indicates the amount of cooking time needed for each type of pasta to reach the ideal texture. It did not vary significantly between the four pasta samples. Russello and Cappelli required cooking times of 10 and 11 min, respectively, while Perciasacchi and Margherito needed slightly longer cooking times of 12 min.

The water absorption index was measured both at the OCT and at the overcooking point for 15 min. Pasta from the Russello and Margherito genotypes had lower water absorption at the OCT—128.17% and 127.86%, respectively—confirming the micro-visco-amylograph results, which showed lower water absorption in Russello than in the other samples. As expected, water absorption increased with overcooking of the pasta, showing a similar trend to the OCT. There were no statistically significant differences between the samples.

With regard to the moisture content of the spaghetti, there were no statistically relevant differences between the samples. They followed a similar trend both before and after cooking. The latter obviously led to an increase in the moisture content of the spaghetti, which was uniform for all samples. This result suggested comparable hydration properties during cooking.

As reported in Table 6, the thickness evaluation of raw spaghetti—which provides information on pasta stability during drying—showed excellent stability for all four tetraploid wheats. Thickness was consistent along their length, which was 2.00 mm for Cappelli and 2.10 mm for the other three varieties. This stability was also confirmed by the evaluation of cooked spaghetti, carried out both at optimum cooking time (OCT) and overcooked at 15 min. The thickness increased uniformly, with Russello and Perciasacchi reaching 3.10 mm and Cappelli and Margherito being slightly above 3.15 mm. The lack of statistically significant differences further confirmed that all samples had almost similar absorption properties.

The texture analysis of cooked pasta by Ta.XT, which differs from the evaluation of pasta “by tasting”, as it tests cold cooked pasta below 50 °C, was performed both at OCT and after 15 min of overcooking. The literature shows that the main qualitative parameters that influence pasta quality include protein content, gluten quantity, and gluten quality, all closely related to the parameters measured by the Ta.XT analyzer (Table S5).

At the OCT, Perciasacchi demonstrated a more consistent and firm texture (10.84 N), followed by Russello and Cappelli. On the other hand, Margherito had the weakest texture, indicating a softer and more delicate pasta. When overcooked for 15 min, all varieties had a reduction in firmness, but Russello and Margherito displayed the most pronounced softness (8.46 N and 8.52 N, respectively). These results suggested that both varieties become significantly softer and less resilient when overcooked, potentially resulting in a mushy texture. In contrast, Cappelli and Perciasacchi remained relatively firm even when overcooked (9.93 N and 8.87 N, respectively). This suggests that these two samples have a more stable texture when cooked for a long time, making them more resilient in terms of texture during preparation.

The cooked pasta was left at room temperature for 5 min. The color was then determined by taking three measurements at three different points on the surface of the cooked pasta. Under the same processing/drying conditions, the four pasta samples gave different colors, suggesting an influence of the wheat cultivar on this parameter (Table 7). The yellow index is one of the quality attributes that particularly characterizes pasta, as well as bakery products, defining their level of consumer acceptability [51]. In particular, raw pasta made from the Perciasacchi cultivar was the most yellow (18.44 b*), in line with observations from semolina color evaluation, while no statistically significant differences were found among the other samples.

With regard to the red index, no statistically significant differences were found among pasta types, except for Cappelli. The values ranged between 1.04 a* and 2.00 a*. In particular, Russello was the reddest (2.00). Lightness values were nearly uniform for all the pasta samples, except for Perciasacchi, which differed significantly.

Cooking caused noticeable reductions in the colorimetric parameters, particularly the red index (a*) and yellow index (b*). These changes may be due to the oxidative activity of the enzyme lipoxygenase, which can lead to the degradation of pigments [52], or they may result from the leaching of pigments into the cooking water, combined with the potential thermal degradation of carotenoids.

Cooking resulted in an increase in lightness across all samples. Perciasacchi exhibited the most significant increase in lightness after cooking (L* = 63.70), becoming the brightest among the cooked samples. It also showed a reduction in the yellow index from 18.44 b* in raw pasta to 13.48 b*, yet it remained higher than the other samples. The superior yellow color of the pasta, even after cooking, represented a distinctive trait tied to its raw wheat composition. The other samples showed similar decreases, with Margherito having the lowest cooked yellow value (12.69 b*).

The a* values decreased markedly upon cooking, shifting to negative values. Russello, initially the reddest sample, showed the largest change, dropping from 2.00 a* to −1.30 a*. This trend was consistent across all samples, reflecting a loss of red pigments during the cooking process.

Regarding b*, color loss during cooking was strongly genotype-dependent. By comparing pre- and post-cooking yellow index values, Russello and Cappelli exhibited a better ability to “retain” their yellow color during cooking, unlike Perciasacchi, which showed significant loss. This genotype–yellow index link has also been observed by other authors in studies conducted on semolina [53].

### 3.7. Sensory Analysis

The sensory attributes of spaghetti assessed through manual handling include three parameters: liveliness, starch release, and firmness, as previously presented in Table 1.

In terms of liveliness (Table 8), all the samples reached scores of 20–30, indicative of a sticky consistency. These values may be closely associated with the starch release (score 40) across samples, which serves as a marker for cooking loss. Russello showed medium firmness (score: 60), while Perciasacchi, Cappelli, and Margherito showed high firmness (all scoring 80). This confirmed the firmness values recorded in the hot viscosity phase of the micro-visco-amylograph (points B and D), which were lower for Russello than for the other three varieties.

**Figure 2:**
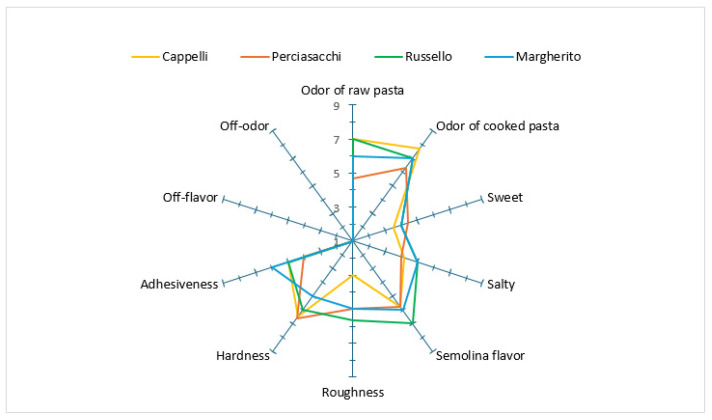
Sensory profile of spaghetti samples obtained from the four tetraploid wheat genotypes.

The sensory evaluation carried out by the judges allowed us to obtain the sensory profile of the pasta samples (Figure 2). These profiles allowed the sensory characteristics of the products to be described objectively by measuring the intensity of specific descriptors.

All the descriptors evaluated were statistically significant with a *p*-value ≤ 0.001.

The intensity of the raw pasta odor was significantly higher in the Cappelli (7.00 ± 0.3) and Russello (6.75 ± 0.3) samples than in Margherito (5.83 ± 0.22) and Perciasacchi (4.67 ± 0.5). The odor intensity of cooked pasta was significantly greater in Cappelli (7.7 ± 0.4) compared to Russello (7.00 ± 0.2), Margherito (6.75 ± 0.3), and Perciasacchi (6.41 ± 0.4). With regard to sweetness, Perciasacchi (4.41 ± 0.55), Russello (4.25 ± 0.22), and Margherito (4.00 ± 0.10) exhibited significantly higher values compared to Cappelli (2.90 ± 0.65). Russello (5.41 ± 0.10) and Margherito (5.25 ± 0.27) were rated as saltier than Cappelli (4.20 ± 0.45) and Perciasacchi (4.00 ± 0.10). Cappelli (5.70 ± 0.45) and Perciasacchi (5.67 ± 0.45) exhibited the lowest semolina flavor intensity compared to Russello (6.58 ± 0.22). The roughness attribute was also significantly lower in Perciasacchi (4.83 ± 0.22) and Cappelli (3.00 ± 0.35) compared to Russello (5.58 ± 0.35).

Hardness was statistically greater in the Perciasacchi (6.60 ± 0.10), Cappelli (6.50 ± 0.12), and Russello (6.33 ± 0.27) pasta samples, while Margherito exhibited the lowest value (5.00 ± 0.10). As regards adhesiveness, Margherito pasta presented the highest value (6.41 ± 0.11), while Perciasacchi pasta showed the significantly lowest value (4.00 ± 0.14).

No off-odors or off-flavors were detected in any of the pasta samples.

### 3.8. Principal Component Analysis (PCA)

As a result of the principal component analysis (PCA), the first two components accounted for 75.0% of the total explained variance (PC1: 42.2%; PC2: 32.7%) (Table S6), effectively distinguishing the four durum wheat varieties in the multidimensional space.

Perciasacchi and Cappelli were located in the positive region of PC1, showing strong positive correlations (>80%)—and thus high values—with flour color indices (red index a* and yellow index b*), dough rheological properties (farinograph stability, dough development time, P/L ratio, and W), starch pasting characteristics (peak, trough, and setback viscosities), flour and gluten quality (falling number and gluten index), and the water absorption index (WAI at OCT).

Conversely, Russello and Margherito, located in the negative region of PC1, were associated with lower values for these parameters.

**Figure 3:**
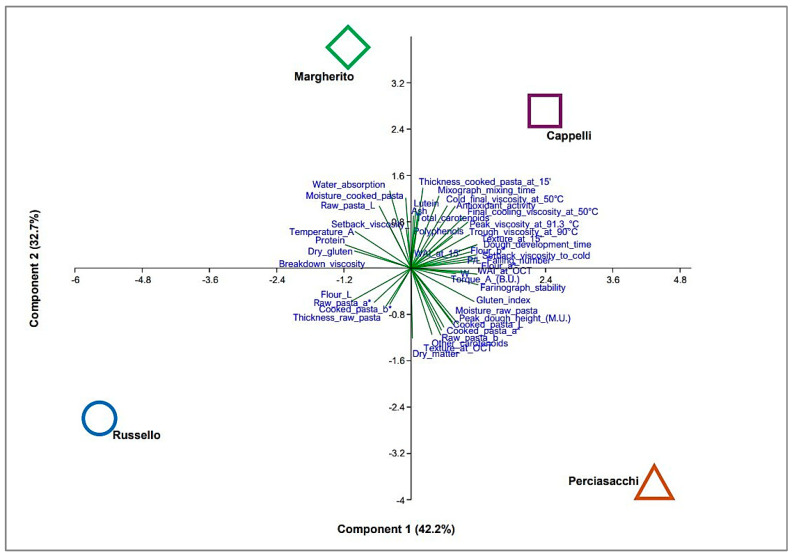
Principal component analysis (PCA) biplot. Vectors represent the loadings of the physical and chemical characteristics of semolina, dough, and both raw and cooked pasta from four traditional and local varieties of durum wheat (Margherito, Russello, Perciasacchi, Cappelli).

PC1 also exhibited strong negative correlations (>−80%) with protein content (% d.m.), flour lightness (L*), dry gluten content (% d.m.), and temperature A (°C) (Figure 3; Table S7).

Margherito and Cappelli, located in the positive region of PC2, showed strong positive correlations (>80%) with cooked pasta thickness (15’, mm), water absorption at 500 B.U. (g/100 g), mixograph mixing time (min), and cooked pasta moisture (%). PC2 also exhibited strong negative correlations (>−80%) with dry matter content (%), other carotenoids (mg/kg), and pasta texture at OCT (N).

Consequently, Perciasacchi and Russello, positioned in the negative region of PC2, exhibited characteristics opposite to those of Margherito and Cappelli (Figure 3; Table S7).

## 4. Conclusions

To the best of our knowledge, this study represents the first comprehensive physico-chemical, rheological, technological, biochemical, and sensory evaluation of ancient Sicilian tetraploid wheat intended for high-quality pasta production.

This study provides new knowledge on the use of ancient tetraploid wheat genotypes for pasta production. Four tetraploid wheats—Russello, Perciasacchi, Margherito, and the historical reference Cappelli—were evaluated for dough rheology, technological and biochemical quality, and the sensory performance of the pasta. Principal component analysis (PCA) highlighted a strong effect of genotype on the characteristics of semolina and pasta.

Perciasacchi and Cappelli showed superior gluten functionality, with higher gluten index values, longer dough development times, and greater strength and stability—key traits for high-quality pasta. Their visco-amylographic profiles also indicated better thermal stability and water retention. Conversely, Russello showed earlier starch gelatinization, lower gel consistency, and poorer cooking performance, suggesting limited suitability for premium pasta.

In terms of biochemical profile, Perciasacchi, Margherito, and Cappelli had higher polyphenol content and antioxidant activity. Margherito was notable for its lutein and carotenoid content, although a significant loss of yellow color was observed after cooking, particularly in Perciasacchi and Margherito.

The sensory analysis confirmed these results: Perciasacchi, Cappelli, and Margherito produced firmer pasta than Russello. Perciasacchi stood out for its balanced texture and flavor, while Cappelli maintained a high-quality sensory profile. Margherito showed less favorable sensory attributes, and Russello exhibited lower firmness and structure.

In conclusion, Perciasacchi emerged as the most promising genotype, combining good technological properties, antioxidant potential, and appealing sensory traits, despite a moderate protein content. Cappelli also confirmed its value as a high-quality reference variety, while the high antioxidant profile of Margherito suggests potential for functional products.

## Figures and Tables

**Table 1:** Specific rating scales for the three parameters.

Firmness	Liveliness	Starch Release
100—very high (very firm)	100—very high (not at all sticky)	100—very low (no starch)
80—high	80—high	80—low
50—medium	50—medium	50—medium
30—low	30—low	30—high
10—very low (very tender)	10—very low (very sticky)	10—very high (large quantity starch)

**Table 2:** Grain characteristics and relative defects of the studied genotypes.

Genotype	Test Weight (kg/hL)	Thousand Kernel Weight (g)	Starchy Kernels (%)	Shrunken Kernels (%)
Cappelli	82.55 ± 0.15b	50.12 ± 0.10c	2.42 ± 0.13b	3.31 ± 0.03b
Margherito	76.45 ± 0.05d	48.81 ± 0.10d	4.03 ± 0.15b	4.66 ± 0.03a
Perciasacchi	83.55 ± 0.13a	62.04 ± 0.13a	4.42 ± 0.10b	0.05 ± 0.06c
Russello	78.26 ± 0.05c	55.15 ± 0.05b	24.33 ± 1.04a	1.80 ± 0.03ab

Different letters in the columns indicate a significant difference (*p* ≤ 0.001) (Tukey).

**Table 3:** Physico-chemical parameters of semolina.

Parameters		Samples		
	Cappelli	Russello	Perciasacchi	Margherito
Particle size distribution (%)				
500 µm	0.72 ± 0.00b	1.02 ± 0.00a	0.94 ± 0.00a	0.67 ± 0.00b
350 µm	20.63 ± 0.00b	9.28 ± 0.00d	32.63 ± 0.00a	18.07 ± 0.00c
250 µm	19.74 ± 0.00b	10.66 ± 0.00c	19.73 ± 0.00b	22.68 ± 0.00a
160 µm	29.05 ± 0.00a	25.19 ± 0.00b	21.74 ± 0.00c	29.12 ± 0.00a
<160 µm	29.87 ± 0.00b	53.84 ± 0.00a	24.97 ± 0.00c	29.46 ± 0.00b
Dry matter (%)	87.89 ± 0.05b	88.18 ± 0.03a	88.15 ± 0.06ab	87.33 ± 0.05c
Ash (% d.m.)	1.21 ± 0.02ab	1.19 ± 0.01b	1.21 ± 0.02ab	1.28 ± 0.01a
Protein content (% d.m.)	13.78 ± 0.15ab	14.30 ± 0.06a	13.54 ± 0.14b	14.11 ± 0.41ab
Dry gluten (% d.m.)	8.78 ± 0.10b	9.85 ± 0.05a	8.92 ± 0.01b	10.02 ± 0.18a
Gluten index	66.81 ± 0.51b	50.95 ± 0.97c	81.26 ± 1.16a	53.33 ± 0.58c
Dry gluten (% d.m.)/Protein content (% d.m.)	0.64 ± 0.01c	0.69 ± 0.01ab	0.66 ± 0.00bc	0.71 ± 0.01a
Lightness (L*)	85.07 ± 0.52b	88.55 ± 0.30a	84.62 ± 0.65b	84.70 ± 0.17b
Red index (a*)	−0.21 ± 0.14ab	−0.61 ± 0.04b	−0.11 ± 0.08a	−0.32 ± 0.15ab
Yellow index (b*)	16.72 ± 0.33a	11.54 ± 0.46b	17.30 ± 0.55a	16.02 ± 1.36ab

Different letters in the columns indicate a significant difference (protein, a* *p* ≤ 0.05; dry gluten, gluten index, L*, b* p ≤ 0.01; dry matter, ash, dry gluten/protein content *p* ≤ 0.001) (Tukey).

**Table 4:** Technological features of durum wheat semolina.

Samples	Farinograph			Alveograph		Falling Number (s)	Mixograph	
	Dough Development Time (min)	Dough Stability (min)	Water Absorption at 500 B.U. * (g/100 g)	W (10^−4^ × J)	P/L		Mixing Time (min)	Peak Dough Height (M.U.) **
Cappelli	4.85 ± 0.07a	13.40 ± 0.28b	61.85 ± 0.49	162.33 ± 3.51ab	7.23 ± 0.27a	539.50 ± 14.85a	5.14 ± 0.20a	5.35 ± 0.35
Russello	2.50 ± 0.14c	2.75 ± 0.07d	60.55 ± 0.64	128.67 ± 3.06c	1.24 ± 0.05c	435.50 ± 7.78b	2.56 ± 0.15b	5.40 ± 0.57
Perciasacchi	4.60 ± 0.14ab	16.95 ± 0.21a	58.80 ± 1.56	166.33 ± 13.43a	6.57 ± 1.11a	524.50 ± 12.02a	3.06 ± 0.15b	6.05 ± 0.21
Margherito	3.90 ± 0.14b	5.65 ± 0.21c	62.50 ± 0.71	136.67.00 ± 2.52bc	3.43 ± 0.06b	476.00 ± 5.66ab	4.42 ± 0.12ab	5.40 ± 0.14

* Brabender units. ** Mixograph units. Different letters in the columns indicate a significant difference (dough development time, falling number, W, P/L: *p* ≤ 0.01; dough stability, mixing time: *p* ≤ 0.001; water absorption, peak dough height *p* = ns) (Tukey).

**Table 5:** Content of carotenoids, total polyphenols, and antioxidant activity of semolina obtained from the four durum wheat genotypes.

Samples	Antioxidant Activity (µmol TE/g d.m.)	Polyphenols (mg GAE/100 g d.m.)	Lutein (mg/Kg d.m.)	Other Carotenoids (mg/kg d.m.)	Total Carotenoids (mg/kg d.m.)
Cappelli	39.28 ± 4.38a	15.84 ± 0.20	7.83 ± 0.14b	0.47 ± 0.00b	8.30 ± 0.14b
Russello	18.40 ± 3.89b	14.93 ± 1.06	6.81 ± 0.19b	0.55 ± 0.02b	7.36 ± 0.21b
Perciasacchi	31.22 ± 0.88ab	14.16 ± 0.22	7.63 ± 0.13b	0.73 ± 0.01a	8.36 ± 0.12b
Margherito	39.33 ± 4.11a	14.56 ± 0.29	10.73 ± 0.25a	0.51 ± 0.01b	11.24 ± 0.25a

TE: trolox equivalents; GAE: gallic acid equivalents. Different letters in the columns indicate a significant difference (antioxidant activity *p* ≤ 0.05; polyphenols *p* = ns; lutein *p* ≤ 0.001) (Tukey).

**Table 6:** Cooking quality and texture evaluation of pasta.

Parameters			Pasta Samples		
		Cappelli	Russello	Perciasacchi	Margherito
Cooking quality	OCT (min)	11.0	10.0	12.0	12.0
	WAI at OCT (%)	136.17 ± 2.91	128.17 ± 2.02	134.63 ± 1.66	127.86 ± 3.07
	WAI at 15’ (%)	147.39 ± 1.96	144.44 ± 1.87	142.25 ± 2.29	141.96 ± 3.01
Spaghetti thickness (mm)	Raw spaghetti	2.00 ± 0.00	2.10 ± 0.00	2.10 ± 0.00	2.10 ± 0.00
	Cooking at OCT	3.10 ± 0.00	3.10 ± 0.00	3.10 ± 0.00	3.10 ± 0.00
	Overcooking at 15’	3.15 ± 0.07	3.10 ± 0.00	3.10 ± 0.00	3.15 ± 0.07
Pasta moisture (%)	Raw pasta	8.38 ± 0.86	8.48 ± 0.12	9.58 ± 0.54	8.57 ± 0.55
	Cooked pasta	61.02 ± 0.09	60.38 ± 0.32	60.33 ± 1.24	62.44 ± 0.28
Texture at OCT (N)		10.36 ± 0.31ab	10.61 ± 0.84ab	10.84 ± 0.17a	8.98 ± 0.09b
Texture overcooking at 15’ (N)		9.93 ± 0.47a	8.46 ± 0.18b	8.87 ± 0.23ab	8.52 ± 0.32b

Different letters in the columns indicate a significant difference (WAI at OCT, WAI at 15’, moisture, thickness *p* = ns; texture at OCT, texture at 15’ *p* ≤ 0.01; water absorption index *p* ≤ 0.001) (Tukey). Other parameters are not statistically processed.

**Table 7:** Colorimetric parameters of raw and cooked pasta.

Samples	Raw Pasta			Cooked Pasta		
	L*	a*	b*	L*	a*	b*
Cappelli	63.11 ± 0.51a	1.04 ± 0.10b	14.95 ± 0.05b	62.55 ± 0.08ab	−1.55 ± 0.06b	14.06 ± 0.25a
Russello	60.58 ± 0.06a	2.00 ± 0.30a	15.93 ± 0.79b	62.02 ± 0.10ab	−1.30 ± 0.03b	14.43 ± 0.01a
Perciasacchi	53.30 ± 1.40b	1.80 ± 0.00a	18.44 ± 0.73a	63.70 ± 0.97a	−0.53 ± 0.02a	13.48 ± 0.59ab
Margherito	61.14 ± 0.76a	1.92 ± 0.18a	15.74 ± 0.35b	61.14 ± 0.01b	−1.30 ± 0.06b	12.69 ± 0.13b

Different letters in the columns indicate a significant difference (raw pasta: *p* ≤ 0.05; cooked pasta: L*, b* p ≤ 0.05; a* p ≤ 0.001) (Tukey).

**Table 8:** Sensory attributes according to ISO 7304-1 standard.

Pasta Samples	Sensory Attributes		
	Liveliness	Starch Release	Firmness
Cappelli	30	40	80
Russello	20	40	60
Perciasacchi	20	40	80
Margherito	30	40	80

## Data Availability

The original contributions presented in the study are included in the article/Supplementary Material. Further inquiries can be directed to the corresponding author.

## Supplementary Materials

The following supporting information can be downloaded at: https://www.mdpi.com/article/10.3390/foods14122050/s1. Figure S1. Representative curve of the thermal profile obtained from visco-amylograph analysis, with the relative points indicated. Figure S2. TA.XT texture diagram. Table S1. Thermal profile used for visco-amylograph analysis. Table S2. Specification of each point of the visco-amylographic curve. Table S3. Descriptors selected to describe the sensory properties of pasta. Table S4. Visco-amylograph parameters of semolina flour samples during heating and cooling phases. Table S5. Parameters measured by the Ta.XT analyzer. Table S6. Principal component analysis (PCA). Eigenvalue and proportion of variance explained by each principal component. Table S7. Principal component analysis (PCA) loadings of the physical and chemical characteristics of flour, dough, and both raw and cooked pasta from four traditional varieties of durum wheat (Russello, Perciasacchi, Cappelli, Margherito).

## References

[1] De Santis M.A., Kosik O., Passmore D., Flagella Z., Shewry P.R., Lovegrove A. (2018). Comparison of the dietary fibre composition of old and modern durum wheat (*Triticum turgidum* spp. durum) genotypes. Food Chem..

[2] Dinelli G., Marotti I., Di Silvestro R., Bosi S., Bregola V., Accorsi M., Di Loreto A., Benedettelli S., Ghiselli L., Catizone P. (2013). Agronomic, nutritional and nutraceutical aspects of durum wheat (Triticum durum Desf.) cultivars under low input agricultural management. Ital. J. Agron..

[3] De Vita P., Di Paolo E., Fecondo G., Di Fonzo N., Pisante M. (2007). No-tillage and conventional tillage effects on durum wheat yield, grain quality and soil moisture content in southern Italy. Soil Till. Res..

[4] Tateo F., Bononi M., Castorina G., Colecchia S.A., De Benedetti S., Consonni G., Geuna F. (2023). Whole-genome resequencing-based characterization of a durum wheat landrace showing similarity to ‘Senatore Cappelli’. PLoS ONE.

[5] Scandurra A., Corinzia S.A., Caruso P., Cosentino S.L., Testa G. (2024). Productivity of Wheat Landraces in Rainfed and Irrigated Conditions under Conventional and Organic Input in a Semiarid Mediterranean Environment. Agronomy.

[6] Palombieri S., Bonarrigo M., Potestio S., Sestili F., Messina B., Russo G., Miceli C., Frangipane B., Genduso M., Delogu C. (2024). Characterization among and within Sicilian Tetraploid Wheat Landraces by Gluten Protein Analysis for Traceability Purposes. Plants.

[7] Fiore M.C., Blangiforti S., Preiti G., Spina A., Bosi S., Marotti I., Mauceri A., Puccio G., Sunseri F., Mercati F. (2022). Elucidating the genetic relationships on the original old Sicilian Triticum Spp. collection by SNP genotyping. Int. J. Mol. Sci..

[8] Cappelli A., Cini E. (2021). Challenges and opportunities in wheat flour, pasta, bread, and bakery product production chains: A systematic review of innovations and improvement strategies to increase sustainability, productivity, and product quality. Sustainability.

[9] Zingale S., Guarnaccia P., Timpanaro G., Scuderi A., Matarazzo A., Bacenetti J., Ingrao C. (2022). Environmental life cycle assessment for improved management of agri-food companies: The case of organic whole-grain durum wheat pasta in Sicily. Int. J. Life Cycle Assess..

[10] Subira J., Peña R.J., Álvaro F., Ammar K., Ramdani A., Royo C. (2014). Breeding progress in the pasta-making quality of durum wheat cultivars released in Italy and Spain during the 20th Century. Crop Pasture Sci..

[11] Visioli G., Giannelli G., Agrimonti C., Spina A., Pasini G. (2021). Traceability of Sicilian durum wheat landraces and historical varieties by high molecular weight glutenins footprint. Agronomy.

[12] Mandolesi S., Cubero Dudinskaya E., Naspetti S., Solfanelli F., Ozturk E., Zanoli R. (2024). Organic Consumers’ Preferences for Dried Pasta Made with Ancient Wheat Varieties. Proceedings of the Italian Association of Agricultural Economists Conference.

[13] Varia F., Macaluso D., Vaccaro A., Caruso P., Guccione G.D. (2021). The adoption of landraces of durum wheat in Sicilian organic cereal farming analysed using a system dynamics approach. Agronomy.

[14] Costanzo A., Bàrberi P. (2014). Functional agrobiodiversity and agroecosystem services in sustainable wheat production. A review. Agron. Sustain. Dev..

[15] Pino A., Russo N., Solieri L., Sola L., Caggia C., Randazzo C.L. (2022). Microbial consortia involved in traditional Sicilian sourdough: Characterization of lactic acid bacteria and yeast populations. Microorganisms.

[16] Suo X., Pompei F., Bonfini M., Mustafa A.M., Sagratini G., Wang Z., Vittadini E. (2023). Quality of wholemeal pasta made with pigmented and ancient wheats. Int. J. Gastron. Food Sci..

[17] Taranto F., Di Serio E., Miazzi M.M., Pavan S., Saia S., De Vita P., D’Agostino N. (2022). Intra-and inter-population genetic diversity of “Russello” and “Timilia” landraces from sicily: A proxy towards the identification of favorable alleles in durum wheat. Agronomy.

[18] De Vita P., Nicosia O.L.D., Nigro F., Platani C., Riefolo C., Di Fonzo N., Cattivelli L. (2007). Breeding progress in morpho-physiological, agronomical and qualitative traits of durum wheat cultivars released in Italy during the 20th century. Eur. J. Agron..

[19] Fiore M.C., Mercati F., Spina A., Blangiforti S., Venora G., Dell’Acqua M., Lupini A., Preiti G., Monti M., Pè M.E. (2019). High-throughput genotype, morphology, and quality traits evaluation for the assessment of genetic diversity of wheat landraces from Sicily. Plants.

[20] Ruisi P., Ingraffia R., Urso V., Giambalvo D., Alfonzo A., Corona O., Settanni L., Frenda A.S. (2021). Influence of grain quality, semolinas and baker’s yeast on bread made from old landraces and modern genotypes of Sicilian durum wheat. Food Res. Int..

[21] Spina A., Guarnaccia P., Canale M., Sanfilippo R., Bizzini M., Blangiforti S., Zingale S., Lo Piero A.R., Allegra M., Sicilia A. (2023). Sicilian Rivet Wheat Landraces: Grain Characteristics and Technological Quality of Flour and Bread. Plants.

[22] AOAC (1995). Official Method 935.25.

[23] (2017). Cereals, Pulses and By-Products—Determination of Ash Yield by Incineration.

[24] (2000). Determination of Crude Protein in Grain and Grain Products for Food and Feed by the Dumas Combustion Principle.

[25] (1995). Gluten Index Method for Assessing Gluten Strength in Durum Wheat (Triticum Durum).

[26] AACC (2000). Approved Methods of Analysis.

[27] Spina A., Scarangella M., Canale M., Sanfilippo R., Giannone V., Summo C., Pasqualone A. (2023). Nutritional features of flour blends composed of durum wheat and lupin. Int. J. Food Sci. Technol..

[28] Vignolini P., Urciuoli S., Heimler D., Romani A. (2018). Carotenoids, polyphenols and antioxidant activity evaluation in stone-grinded wheat semolina. J. Health Sci..

[29] Singleton V.L., Orthofer R., Lamuela-Raventos R.M. (1999). Analysis of total phenols and other oxidation substrates and antioxidant by means of Folin-Ciocalteu reagent. Method Enzymol..

[30] Cao G., Alessio H.M., Cutler R.G. (1993). Oxygen-radical absorbance capacity assay for antioxidants. Free Radic. Biol. Med..

[31] Ou B., Hampsch-Woodill M., Prior R. (2001). Development and validation of an improved oxygen radical absorbance capacity assay using fluorescein as the fluorescent probe. J. Agric. Food Chem..

[32] (1995). Durum Wheat and Semolina—Determination of Rheological Properties Using an Alveograph.

[33] (2009). Wheat, Rye and Their Flours, Durum Wheat and Durum Wheat Semolina—Determination of the Falling Number According to Hagberg-Perten.

[34] Ali R., Khan M.S., Sayeed S.A., Ahmed R., Saeed S.M.G., Mobin L. (2014). Relationship of damaged starch with some physicochemical parameters in assessment of wheat flour quality. Pak. J. Bot..

[35] Mariotti M., Zardi M., Lucisano M., Pagani M.A. (2005). Influence of the heating rate on the pasting properties of various flours. Starch-Stärke.

[36] Murray J.C., Kiszonas A.M., Morris C.F. (2017). Pasta production: Complexity in defining processing conditions for reference trials and quality assessment methods. Cereal Chem..

[37] (2016). Durum Wheat Semolina and Alimentary Pasta—Estimation of Cooking Quality of Alimentary Pasta by Sensory Analysis. Part 1: Reference Method.

[38] Bokić J., Škrobot D., Tomić J., Šeregelj V., Abellán-Victorio Á., Moreno D.A., Ilić N. (2022). Broccoli sprouts as a novel food ingredient: Nutritional, functional and sensory aspects of sprouts enriched pasta. LWT.

[39] (2014). Sensory Analysis—General Guidance for the Design of test Rooms. Amendment 1. 2nd ed. Technical Committee, ISO/TC 34/SC 12 Sensory Analysis.

[40] (2023). Sensory Analysis—Selection and Training of Sensory Assessors.

[41] (2008). Alimentary Pasta Produced from Durum Wheat Semolina—Estimation of Cooking Quality by Sensory Analysis—Part 2: Routine Method.

[42] Pagliarini E., Società Italiana di Scienze Sensoriali (2012). Atlante Sensoriale dei Prodotti Alimentari Book.

[43] Hammer Ø., Harper D.A.T., Ryan P.D. (2021). PAST: Paleontological statistics software package for education and data analysis. Palaeontol. Electron..

[44] Venora G., Grillo O., Saccone R. (2009). Quality assessment of durum wheat storage centres in Sicily: Evaluation of vitreous, starchy and shrunken kernels using an image analysis system. J. Cereal Sci..

[45] Dziki D., Krajewska A., Findura P. (2024). Particle Size as an Indicator of Wheat Flour Quality: A Review. Processes.

[46] Presidential Decree n° 187. 2001. Regulation for the Revision of Laws Concerning the Production and Sale of Milling Products and Pasta, Pursuant to Article 50 of Law N° 146, Dated 22 February 1994. Official Journal n. 117. https://www.gazzettaufficiale.it/atto/serie_generale/caricaDettaglioAtto/originario?atto.dataPubblicazioneGazzetta=2001-05-22&atto.codiceRedazionale=001G0242&elenco30giorni=false.

[47] Giunta F., Bassu S., Mefleh M., Motzo R. (2020). Is the technological quality of old durum wheat cultivars superior to that of modern ones when exposed to moderately high temperatures during grain filling?. Foods.

[48] Cecchini C., Bresciani A., Menesatti P., Pagani M.A., Marti A. (2021). Assessing the rheological properties of durum wheat semolina: A review. Foods.

[49] Carpentieri S., Larrea-Wachtendorff D., Donsì F., Ferrari G. (2022). Functionalization of pasta through the incorporation of bioactive compounds from agri-food by-products: Fundamentals, opportunities, and drawbacks. Trends Food Sci. Technol..

[50] Balli D., Cecchi L., Pieraccini G., Innocenti M., Benedettelli S., Mulinacci N. (2022). What’s new on total phenols and γ-oryzanol derivatives in wheat? A comparison between modern and ancient varieties. J. Food Compos. Anal..

[51] Oduro-Obeng H., Fu B.X., Beta T. (2021). Influence of cooking duration on carotenoids, physical properties and in vitro antioxidant capacity of pasta prepared from three Canadian durum wheat cultivars. Food Chem..

[52] Carrera A., Echenique V., Zhang W., Helguera M., Manthey F., Schrager A., Picca A., Cervigni G., Dubcovsky J. (2007). A deletion at the Lpx-B1 locus is associated with low lipoxygenase activity and improved pasta color in durum wheat (*Triticum turgidum* ssp. durum). J. Cereal Sci..

[53] Fu B.X., Schlichting L., Pozniak C.J., Singh A.K. (2013). Pigment loss from semolina to dough: Rapid measurement and relationship with pasta colour. J. Cereal Sci..

