# Supplementary material for: The Potential of Ancient Sicilian Tetraploid Wheat in High-Quality Pasta Production: Rheological, Technological, Biochemical, and Sensory Insights
Source: Foods. 2025 Jun 11;14(12):2050. doi: 10.3390/foods14122050 (PMC12191580; doi:10.3390/foods14122050)
Supplement: Supplementary file 1 [file foods-14-02050-s001.zip › Table S1.pdf]

Table S1. Thermal profile used for visco-amylograph analysis.

Segment	Slope (°C/min)	Temperature (° C)	Hold time (HH:MM:SS)	Ramp time (HH:MM:SS)
0	0.0	30	00:00:00	
1	+ 4.0	90		00:15:00
2	0.0	90	00:07:00	
3	- 4.0	50		00:10:00
4	0.0	50	00:05:00	
