# Supplementary material for: The Potential of Ancient Sicilian Tetraploid Wheat in High-Quality Pasta Production: Rheological, Technological, Biochemical, and Sensory Insights
Source: Foods. 2025 Jun 11;14(12):2050. doi: 10.3390/foods14122050 (PMC12191580; doi:10.3390/foods14122050)
Supplement: Supplementary file 1 [file foods-14-02050-s001.zip › Table S2.pdf]

Table S2. Specification of each point of the visco-amylographic curve.

**Point A**	Start of gelatinization	Easy hydration and consequent gelatinization of the more accessible starch granules, such as starch damaged during the milling process;
**Point C**	Rapid increase of gelatinization	Complete hydration and subsequent gelatinization of all the starch granules. It determines the rapid increase in viscosity
**Point B**	Peak viscosity (PV) and Peak time (tP)	The point of maximum hot viscosity and the time taken to reach it, respectively
**Point D**	Trough viscosity (TV)	It measures the stability of the gel in hot stationary phase and before the point at which cooling begins. It is linked to the amylase activity and the release of water caused by the bursting/breaking of the starch granules
**Value B-D**	Breakdown viscosity (BV)	It indicates the degree of weakening of the gel, calculated from the difference between the Peak viscosity (PV) value and the Trough viscosity (TV) value.
**Point E**	Final cooling viscosity (FV_50)	It measures the retrogradation of the starch during the cooling phase up to 50 °C.
**Value E-D**	Set-back viscosity (SV1)	It indicates the increase in viscosity caused by cooling and the reorganization of the starch structure, calculated from the difference between the "viscosity at the end of cooling at 50°C" and the "minimum hot viscosity value (TV)".
**Point F**	Final cold viscosity (FV.cold)	It indicates the stability of the cold gel, obtained during the stationary cooling phase at the end of the test.
**Value E-F**	Setback viscosity to cold (SV2)	It indicates the weakening of the cold gel over time due to the release of water. It is calculated from the difference between the viscosity at the end of cooling to 50°C and the viscosity value at the end of the test.
