# Supplementary material for: The Potential of Ancient Sicilian Tetraploid Wheat in High-Quality Pasta Production: Rheological, Technological, Biochemical, and Sensory Insights
Source: Foods. 2025 Jun 11;14(12):2050. doi: 10.3390/foods14122050 (PMC12191580; doi:10.3390/foods14122050)
Supplement: Supplementary file 1 [file foods-14-02050-s001.zip › Table S3.pdf]

**Table S3.** Descriptors selected to describe the sensory properties of pasta

| SENSORY EVALUATION OF PASTA | | | |
|---|---|---|---|
| Olfactory sensations (orthonasal perception) | | Pasta | Characteristic olfactory intensity of raw semolina pasta |
| | | Cooked | Characteristic olfactory intensity of cooked semolina pasta |
| | | Off-odor | Unpleasant odors |
| Flavor (sensory evaluation taste) | Taste sensations | Sweet | Taste sensation produced by sweet substances (e.g. sucrose) perceived during chewing |
| | | Salty | Taste sensation produced by salts (e.g. sodium chloride) perceived during chewing |
| | | Off-flavor | Unpleasant flavor |
| | Olfactory sensations (retronasal perception) | Semolina | Olfactory-gustatory sensation associated with cooked semolina pasta |
| Texture and other tactile sensations | | Roughness | Describes the degree of irregularity of the surface of the pasta |
| | | Hardness | Force required to compress pasta between the incisors until it breaks |
| | | Adhesiveness | Degree of adhesion of the pasta to the teeth (with chewing) or to the palate (compression on the palate). It is an indicator of the stickiness |
