# Supplementary material for: The Potential of Ancient Sicilian Tetraploid Wheat in High-Quality Pasta Production: Rheological, Technological, Biochemical, and Sensory Insights
Source: Foods. 2025 Jun 11;14(12):2050. doi: 10.3390/foods14122050 (PMC12191580; doi:10.3390/foods14122050)
Supplement: Supplementary file 1 [file foods-14-02050-s001.zip › Table S4.pdf]

Table S4. Visco-Amylograph parameters of semolina flour samples during heating and cooling phases

**Samples**	**Beginning of gelatinization**			**Rapid increase of gelatinization**			**Peak viscosity (PV)**	**Trough viscosity (TV)**	**Breakdown viscosity (BV)**	**Final cooling viscosity (FV.50)**	**Setback viscosity (SV1)**	**Cold final viscosity (FV.cold)**	**Setback viscosity to cold (SV2)**
	**Point A**			**Point C**			**Point B 15' min 91,3°C**	**Point D 22' min. 90°C**	**Value BD**	**Point E 32' min 50°C**	**Value ED**	**Point F 37' min 50°C**	**Value EF**
	Time (mm.ss)	Temp. (°C)	Torque (UB)	Time (mm.ss)	Temp. (°C)	Torque (UB)	Torque (UB)	Torque (UB)	Torque (UB)	Torque (UB)	Torque (UB)	Torque (UB)	Torque (UB)
	t'A	T°C.A	UB.A	t'C	T°C.C	UB.C	UB.B	UB.D	UB.BD	UB.E	UB.ED	UB.F	UB.EF
Margherito	8:08 ±0:00	64,2 ±0,1	15,5 ±0,7	13:30 ±0:00	83,5 ±0,1	50 ±0,0	840±2,0	734±4,0	106±2,0	1242 ±1,0	508 ±3,0	1070 ±6,5	173 ±5,5
Cappelli	7:56 ±0:03	62,4 ± 0,0	13,0 ±1,4	12:50 ±0:00	81,1 ±0,1	48 ±0,0	853±5,0	720±2,5	134±2,5	1263 ±4,5	543 ±2,0	1039 ±1,0	224 ±3,5
Perciasacchi	7:58 ±0:00	61,9 ± 1,1	17,0 ±0,7	13:00 ±0:00	82,5 ±0,0	49 ±0,0	831±7,5	735±13,0	96±5,5	1218 ±3,0	483 ±16,0	1017 ±0,5	202 ±3,5
Russello	8:11 ±0:01	63,7 ± 0,1	12,0 ±2,8	12:00 ±0:00	78,0 ±0,0	48 ±0,0	741±4,0	591±2,5	151±1,5	1114 ±4,5	523 ±2,0	958 ±2,0	156 ±2,5
