# Supplementary material for: The Potential of Ancient Sicilian Tetraploid Wheat in High-Quality Pasta Production: Rheological, Technological, Biochemical, and Sensory Insights
Source: Foods. 2025 Jun 11;14(12):2050. doi: 10.3390/foods14122050 (PMC12191580; doi:10.3390/foods14122050)
Supplement: Supplementary file 1 [file foods-14-02050-s001.zip › Table S5.pdf]

**Table S5.** Parameters measured by the Ta.XT analyzer

| Texture | Cappelli | Russello | Perciasacchi | Margherito |
|---|---|---|---|---|
| Peak Force OCT (N) | 10,36±0,30ab | 10,61±0,84ab | 10,84±0,17a | 8,98±0,09b |
| Area OCT (kg.sec) | 0,73±0,03ab | 0,81±0,06a | 0,78±0,02ab | 0,59±0,01b |
| Time OCT (s′) | 2,24±0,02ab | 2,21±0,07b | 2,28±0,02ab | 2,32±0,01a |
| Peak Force 15′ (N) | 9,93±0,47a | 8,46±0,18b | 8,87±0,23ab | 8,52±0,32b |
| Area 15′ (kg.sec) | 0,69±0,02a | 0,60±0,01ab | 0,66±0,01ab | 0,57±0,02b |
| Time 15′ (s′) | 2,33±0,01ab | 2,28±0,01b | 2,27±0,03b | 2,36±0,01a |

Different letters in the columns indicate a significant difference: Area $p \leq 0.001$; Peak force $p \leq 0.01$; Time at OCT $p \leq 0.05$; Area 15′ $p \leq 0.001$; Peak force (N) 15′, Time 15′ $p \leq 0.01$ (Tukey).
