# Supplementary material for: The Potential of Ancient Sicilian Tetraploid Wheat in High-Quality Pasta Production: Rheological, Technological, Biochemical, and Sensory Insights
Source: Foods. 2025 Jun 11;14(12):2050. doi: 10.3390/foods14122050 (PMC12191580; doi:10.3390/foods14122050)
Supplement: Supplementary file 1 [file foods-14-02050-s001.zip › Table S6.pdf]

Table S6. Principal Component Analysis (PCA). Eigenvalue and proportion of variance explained by each principal component.

PC	Eigenvalue	% variance	Cumulative variance %
1	18,5874	42,244	42,244
2	14,3927	32,711	74,955
3	11,0199	25,045	
