# Supplementary material for: The Potential of Ancient Sicilian Tetraploid Wheat in High-Quality Pasta Production: Rheological, Technological, Biochemical, and Sensory Insights
Source: Foods. 2025 Jun 11;14(12):2050. doi: 10.3390/foods14122050 (PMC12191580; doi:10.3390/foods14122050)
Supplement: Supplementary file 1 [file foods-14-02050-s001.zip › Table S7.pdf]

Table S7. Principal component analysis (PCA) loadings of the physical and chemical characteristics of flours, dough, and both raw and cooked pasta from four traditional varieties of durum wheat (Russello, Perciasacchi, Cappelli, Margherito).

	Axis 1	Axis 2	Axis 3	Axis 4
Dry matter (%)	0,01881	-0,8629	-0,505	2,83E-14
Ash (% d.m.)	0,03457	0,6419	0,766	1,46E-15
Protein (% d.m.)	-0,9523	0,2865	0,105	4,51E-15
Flour lightness (L*)	-0,8505	-0,4014	-0,3399	-1,45E-15
Flour red index (a*)	0,9872	0,09307	0,1293	3,81E-16
Flour yellow index (b*)	0,9411	0,2836	0,1843	1,29E-15
Dry gluten (% d.m.)	-0,8184	0,2129	0,5337	1,58E-15
Gluten index	0,9111	-0,4105	-0,0359	-9,94E-16
Antioxidant activity (μmol TE/g d.m.)	0,6354	0,7599	0,1373	1,43E-15
Polyphenols (mg GAE/100 g d.m.)	-0,0715	0,4976	-0,8645	-2,47E-15
Lutein (mg/Kg)	0,06904	0,7334	0,6763	2,93E-15
Other carotenoids (mg/Kg)	0,4276	-0,8244	0,3709	-5,98E-16
Total carotenoids (mg/Kg)	0,09914	0,6926	0,7144	2,66E-15
Dough development time (min)	0,9532	0,2892	-0,08854	3,51E-16
Farinograph stability (min)	0,9684	-0,2045	-0,1429	-6,65E-16
Water abstorption at 500 B.U. (g/100 g)	-0,3097	0,9495	-0,04979	3,56E-15
W (10-4 x J)	0,9537	-0,0742	-0,2914	-8,11E-16
P/L	0,9543	0,1256	-0,2713	-3,07E-16
Falling number (s)	0,95	0,1638	-0,2658	-4,61E-16
Mixograph mixing time (min)	0,4033	0,8864	-0,2274	1,64E-15
Peak dough height (M.U.)	0,6134	-0,6995	0,3667	-1,15E-15
WAI at OCT (%)	0,8217	-0,04592	-0,5681	-5,20E-15
WAI at 15' (%)	0,01514	0,2519	-0,9676	2,22E-15
Thickness raw pasta (mm)	-0,3699	-0,4767	0,7975	-2,26E-15
Thickness cooked pasta at 15' (mm)	0,17	0,9838	-0,05701	2,20E-15
Moisture raw pasta (%)	0,5988	-0,6641	0,4477	-8,45E-16
Moisture cooked pasta (%)	-0,07793	0,8624	0,5003	9,51E-16
Texture at OCT (N)	0,3028	-0,8167	-0,4912	-3,65E-15
Texture at 15' (N)	0,5977	0,3865	-0,7024	-3,37E-16
Lightness raw pasta (L*)	-0,4606	0,7623	-0,4547	1,14E-15
Red index raw pasta (a*)	-0,5328	-0,4224	0,7333	6,33E-16
Yellow index raw pasta (b*)	0,4372	-0,7639	0,4746	9,66E-16
Lightness cooked pasta (L*)	0,6795	-0,6898	-0,2499	5,36E-15
Red index cooked pasta (a*)	0,4732	-0,7249	0,5006	-8,48E-16
Yellow index cooked pasta (b*)	-0,3076	-0,4446	-0,8413	-2,67E-15
Temperature A (°C)	-0,8119	0,4502	0,3716	-7,40E-17
Torque A (B.U.)	0,6492	-0,06832	0,7575	1,16E-15
Peak viscosity at 91.3 °C (B.U.)	0,8173	0,5653	0,1117	1,45E-15
Trough viscosity at 90°C (B.U.)	0,845	0,4114	0,3417	1,57E-15

Breakdown viscosity (B.U.)	-0,6916	0,001921	-0,7223	-1,04E-15
Final cooling viscosity at 50°C (B.U.)	0,768	0,6375	0,06181	1,42E-15
Setback viscosity	-0,3236	0,5327	-0,782	-6,09E-17
Cold final viscosity at 50°C (B.U.)	0,5216	0,7658	0,3761	2,57E-15
Setback viscosity to cold (B.U.)	0,8692	0,2024	-0,4512	-5,22E-16
