# Supplementary figures and images for: The Potential of Ancient Sicilian Tetraploid Wheat in High-Quality Pasta Production: Rheological, Technological, Biochemical, and Sensory Insights
Source: Foods. 2025 Jun 11;14(12):2050. doi: 10.3390/foods14122050 (PMC12191580; doi:10.3390/foods14122050)

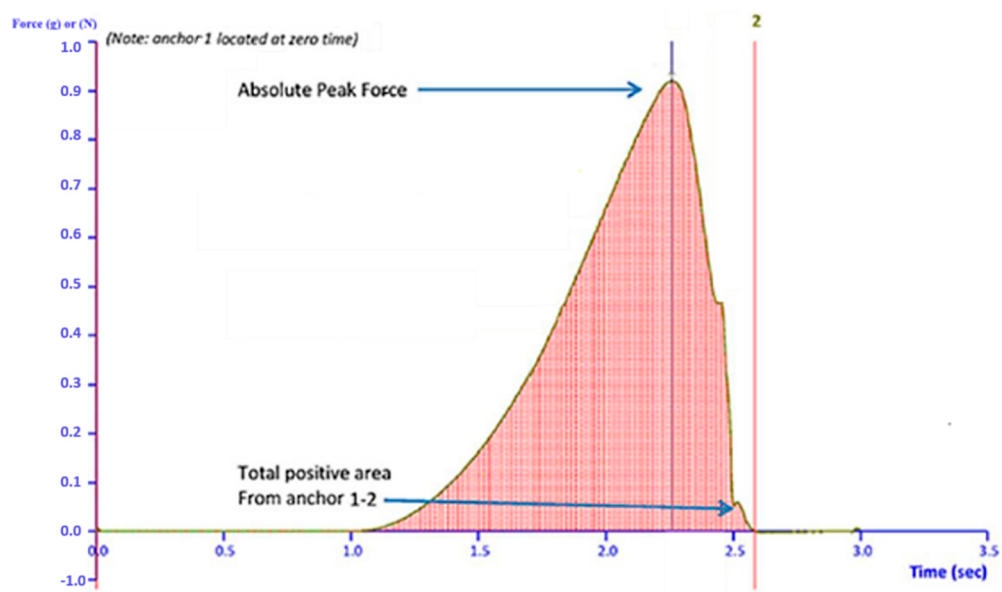

Figure S2. TA.XT texture diagram

Supplement: Supplementary file 1 [file foods-14-02050-s001.zip › Figure S2.pdf]
